# Imaging-Guided Micromachines: Towards Intelligent Systems

**DOI:** 10.3390/mi13112016

**Published:** 2022-11-18

**Authors:** Qianqian Wang

**Affiliations:** Jiangsu Key Laboratory for Design and Manufacture of Micro-Nano Biomedical Instruments, School of Mechanical Engineering, Southeast University, Nanjing 211000, China; qqwang@seu.edu.cn

Micromachines with controllable motion, deformation, and collective behaviors provide advanced methods for performing tasks that traditional machines have difficulty completing thanks to the development of small-scale robotics, nanotechnology, biocompatible materials, and imaging techniques. Compared with machines at the macroscale, micromachines are “tiny” enough to reach confined spaces, allowing localized manipulation in narrow regions, while they are “large” enough to deliver cells, drugs, energy, and fluids in a targeted manner.

As a rapidly growing interdisciplinary research field, the development of micromachine systems offers various research opportunities in the design, control, localization, and functionalization of micromachines, aiming to achieve required tasks in different scenarios (Figure 1). When micromachines are joined in the system, imaging feedback plays an essential role in providing state feedback of both the micromachine and the environment and monitoring the machine–machine and machine–environment interactions. Therefore, the topic editors launched this Special Issue titled Imaging-Guided Intelligent Micromachines, providing an interdisciplinary point of view for designing intelligent micromachine systems.

Integrating medical imaging systems into micromachine systems is a critical step for transferring micromachines from laboratory scenarios to real medical applications. In paper [1], Zhang proposes that although micromachines introduce revolutionary improvements to minimally invasive diagnostics and therapeutics, the existing research still limits over-simplified laboratory environments with unrealistic working conditions. The complex and unstructured biological environments, such as bio-fluids, bloodstream, and narrowed lumens, will certainly affect the performance of micromachines. The integration of medical imaging modalities provides feedback that direct line-of-sight imagin cannot qualify, enabling the in vivo control of micromachines. To design an imaging-guided intelligent system, the other components or sub-systems of a micromachine system should at least be compatible with medical imaging modalities. This requires collaboration among scientists, engineers, and medical practitioners. This review also sheds light on the potential application of next-generation techniques in imaging-guided micromachines and discusses the challenges and opportunities of medical trials.

System integration is a key issue in building intelligent micromachine systems. Integrating medical imaging with a control system enables accurate micromachine navigation in an autonomous manner. Zhao et al., point out that the low precision and efficiency of manual control can be tackled by system integration between autonomous actuation technology and medical imaging [2]. The efficiency of actuation and imaging affects the performance of autonomous navigation in vivo. The interference among sub-systems and the micromachine itself also challenges the system’s integration level. The registration of the workspace can increase the system’s performance, such as the diameter of lumen and bloodstream distribution; however, this may bring new errors to the navigation process. Considering the tiny size of micromachines, the navigation velocity is insufficient for full-body-scale medical tasks. Recent studies indicate that targeted and localized applications can be implemented by assisting tethered medical tools. For example, catheters can be used to inject micromachines before reaching tortuous regions that traditional catheters find difficult in achieving.

This Special Issue includes fundamental research that shows promising application potentials in using micromachines for manipulation. The rapid response and high repeatability features of piezoelectric actuators enable broad applications in micromanipulation tasks. However, complex hysteresis decreases the modeling and control precision in dynamic applications. To tackle this challenge, Zhou et al., present an alternative digitized representation of the modified Prandtl–Ishlinskii hysteresis model. The asymmetric hysteresis behavior of piezoelectric actuators is analyzed by a dead-zone operator hysteresis model [3]. The digitized representation also avoids inversion calculations. The modified Prandtl–Ishlinskii hysteresis model is experimentally validated, showing applications in micromachine systems. While various fabrication methods have been demonstrated and applied to the design and functionalization of micromachines, researchers are developing new strategies to meet the three-dimensional nanostructure construction requirements of micromachines. Yu et al., propose a novel silver-filled nanotube fabrication method that allows the interconnection of materials at the nanometer scale [4]. Current nanotube filling and direct synthesis techniques usually involve obstacles with low filling rates and discontinuous metalcores with limited filling lengths. In this paper, the dominant forces (electromigration and thermal gradient force) are investigated, enabling the continued outflow of the internal silver. Results show the controllable melting and ultrafine flow of the encapsulated silver at a femtogram level (0.83 fg/s), demonstrating potential applications in fabricating micromachines with nanoelectronic components.

Micromachines and their intelligent systems are rapidly growing research fields [5,6,7,8,9,10]. The guest editors hope that the articles and reviews in this Special Issue could attract researchers from different disciplines and promote more interdisciplinary cooperation. We believe that the advancement in imaging-guided intelligent micromachine systems will accelerate the growth of this emerging field.

## Figures and Tables

**Figure 1:**
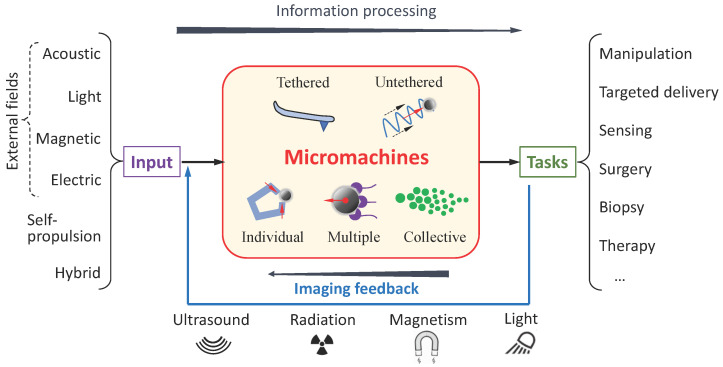
Schematic illustration of a typical imaging-guided micromachine system.

## Data Availability

Not applicable.
